# Supplementary figures and images for: p27Kip1 promotes invadopodia turnover and invasion through the regulation of the PAK1/Cortactin pathway
Source: eLife. 2017 Mar 13;6:e22207. doi: 10.7554/eLife.22207 (PMC5388532; doi:10.7554/eLife.22207)

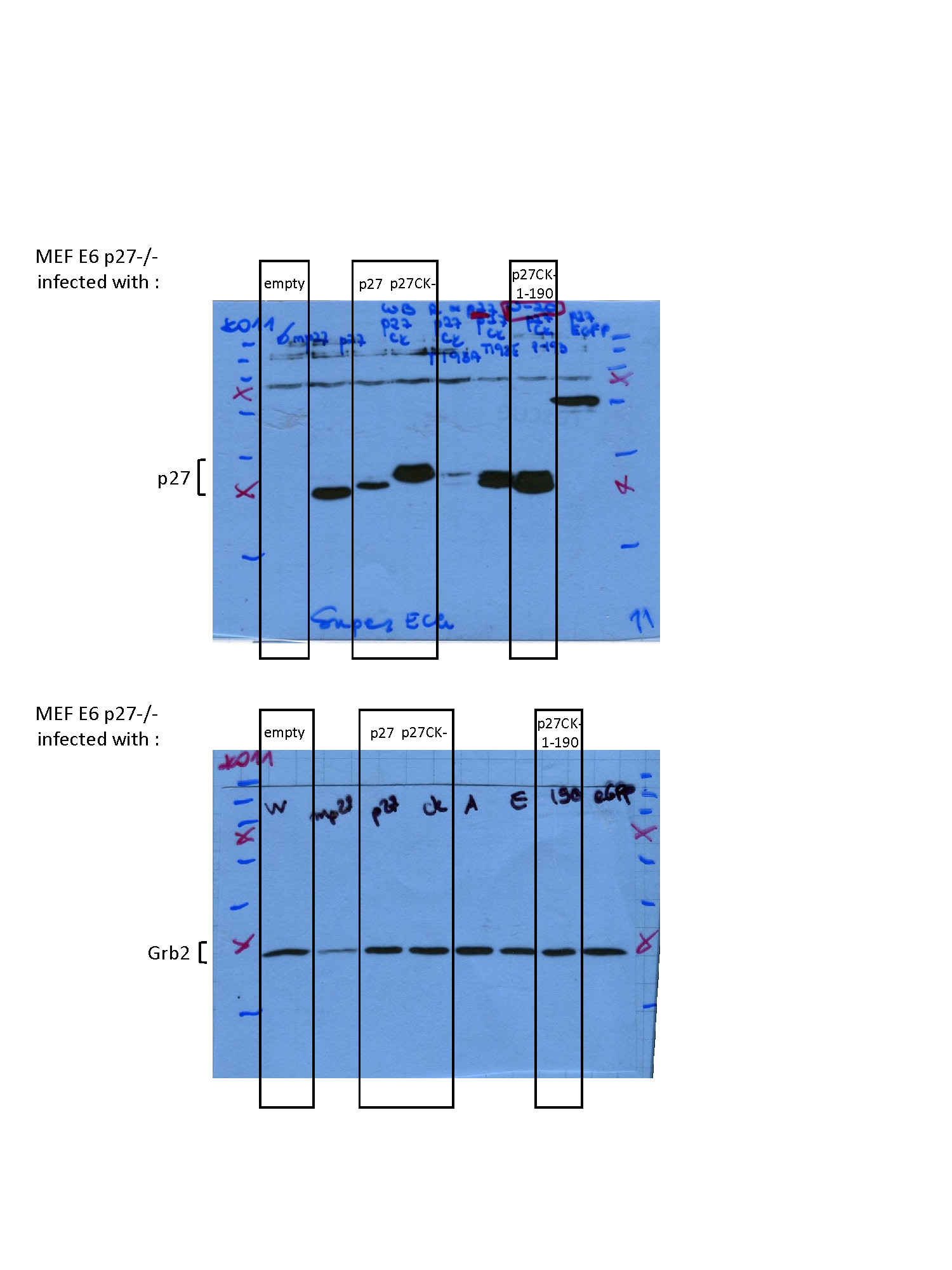

Supplement: Figure 3—source data 3. — DOI: http://dx.doi.org/10.7554/eLife.22207.012 [file elife-22207-fig3-data3.jpg]
